# Unveiling the role of CaMKII in retinal degeneration: from biological mechanism to therapeutic strategies

**DOI:** 10.1186/s13578-024-01236-2

**Published:** 2024-05-09

**Authors:** Yuxin Sun, Mengyu Hao, Hao Wu, Chengzhi Zhang, Dong Wei, Siyu Li, Zongming Song, Ye Tao

**Affiliations:** 1 Department of Ophthalmology, Henan Eye Hospital, Henan Provincial People’s Hospital, People’s Hospital of Zhengzhou University, Zhengzhou, 450003 China; 2 College of Medicine, Zhengzhou University, Zhengzhou, 450001 China

**Keywords:** Therapeutics, CaMKII, Neurodegeneration, Photoreceptor, Diabetic retinopathy

## Abstract

Ca^2+^/calmodulin-dependent protein kinase II (CaMKII) is a family of broad substrate specificity serine (Ser)/threonine (Thr) protein kinases that play a crucial role in the Ca^2+^-dependent signaling pathways. Its significance as an intracellular Ca^2+^ sensor has garnered abundant research interest in the domain of neurodegeneration. Accumulating evidences suggest that CaMKII is implicated in the pathology of degenerative retinopathies such as diabetic retinopathy (DR), age-related macular degeneration (AMD), retinitis pigmentosa (RP) and glaucoma optic neuropathy. CaMKII can induce the aberrant proliferation of retinal blood vessels, influence the synaptic signaling, and exert dual effects on the survival of retinal ganglion cells and pigment epithelial cells. Researchers have put forth multiple therapeutic agents, encompassing small molecules, peptides, and nucleotides that possess the capability to modulate CaMKII activity. Due to its broad range isoforms and splice variants therapeutic strategies seek to inhibit specifically the CaMKII are confronted with considerable challenges. Therefore, it becomes crucial to discern the detrimental and advantageous aspects of CaMKII, thereby facilitating the development of efficacious treatment. In this review, we summarize recent research findings on the cellular and molecular biology of CaMKII, with special emphasis on its metabolic and regulatory mechanisms. We delve into the involvement of CaMKII in the retinal signal transduction pathways and discuss the correlation between CaMKII and calcium overload. Furthermore, we elaborate the therapeutic trials targeting CaMKII, and introduce recent developments in the zone of CaMKII inhibitors. These findings would enrich our knowledge of CaMKII, and shed light on the development of a therapeutic target for degenerative retinopathy.

## Background

Retinal degeneration is a large cluster of retinopathies that characterized by the progressive photoreceptor death and vision loss. These degenerative retinopathies, such as the age-related macular degeneration (AMD), diabetic retinopathy (DR), retinitis pigmentosa (RP) and glaucoma optic neuropathy, acts as the leading cause of blindness in the world [1]. In view of the poor regenerative capacity of retinal neurons, retinal degeneration is considered as an refractory process that causes irreversible visual impairments. In ophthalmological practice, current guidance on the treatment of retinal degeneration is limited. Laser photocoagulation, vitrectomy and various microsurgical therapies act as the main therapeutic method for degenerative retinopathy. However, these treatments are approaches highly invasive and complicated. Therefore, new therapies with satisfactory safety and efficiency are in urgent need.

CaMKII, a protein kinase as an intracellular Ca^2+^ sensor, is garnering increasing amounts of research interest. Protein kinases can alter the conformation of proteins through phosphorylation at crucial sites. Unlike several kinases which solely regulate the function of a few proteins, CaMKII has a broad spectrum of substrate specificity and is therefore involved in modulating numerous functional responses. Typically, CaMKII is most abundantly expressed in neural tissue and acts as a mediator within the neuronal network. For instance, CaMKII is essential for the coupling strength of electrical synapses in retinal neurons, thereby modulating scotopic vision and luminance adaptation [2]. Moreover, CaMKII exerts its effect on long-term potentiation and memory consolidation via binding to the N-methyl-D-aspartate (NMDA)-type glutamate receptor [3]. In particular, the CaMKII activation facilitated by external Ca^2+^ via presynaptic pathways plays a determinant role in neurotransmitter exocytosis [4]. Accordingly, CaMKII may contribute to the pathologic progression of neurodegeneration. A previous study has shown that CaMKII promotes the neurofibrillary degeneration in Alzheimer’s disease [5]. CaMKII is also involved in other activities of non-neuronal cells, such as the cancer cell proliferation and dissemination, insulin secretion, the cell cycle and differentiation [6, 7]. Particularly in the heart, CaMKII has emerged as a validated pathologic signal in several processes related to cardiac dysfunction, such as myocardial infarction, ischemia–reperfusion (I/R) injury, heart failure, arrhythmias and adverse myocardial remodeling [8, 9]. In this context, unveiling the intricate functions of CaMKII may open new avenues for understanding the pathological mechanisms of neurodegeneration and identifying potential therapeutic targets. Thus far, several critical questions remain enigmatic: (1) What is the exact role signaling pathways of CaMKII in retinal degeneration? (2) Will approaches targeting CaMKII provide potential therapeutic strategies for retinal degeneration? (3) How to develop more selective and efficient CaMKII inhibitors? Through addressing these complex and unresolved questions, we can gain deeper insights into the intricate roles of CaMKII in retinal degeneration and pave the way for developing novel therapeutics. In this review, we summarize recent findings on the CaMKII mediated effects in the retinal degeneration as well as its signaling pathways. Furthermore, we elaborate the therapeutic trials targeting CaMKII, and introduce recent developments in the zone of CaMKII inhibitors. These findings would enrich our knowledge of CaMKII, and hight the possibility to identify a pharmacological target for degenerative retinopathy.

## Overview of CaMKII

**Fig. 1:**
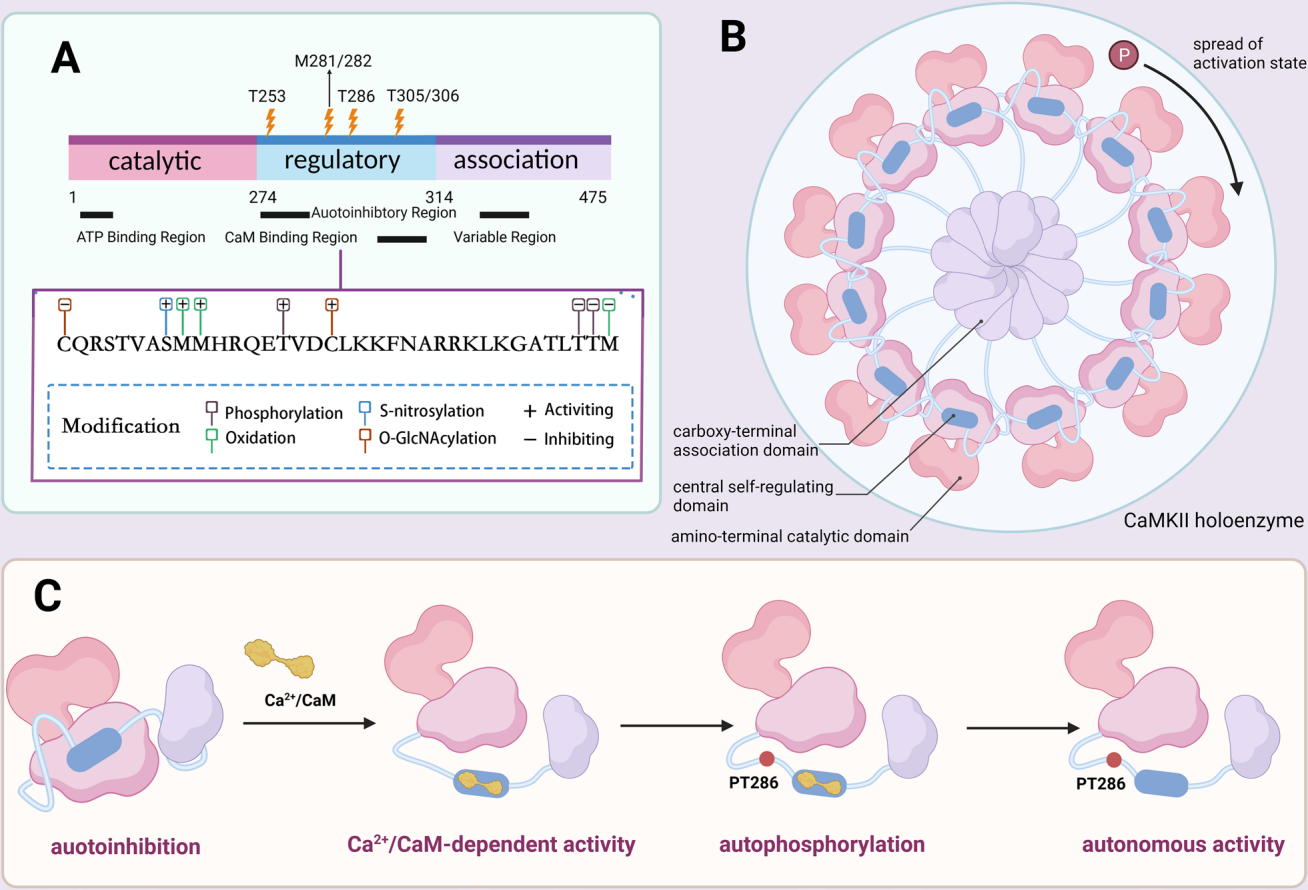
Diagram of CaMKII structure and activation mechanisms. **A** The sequence of CaMKII includes the amino-terminal catalytic domain, the central self-regulating domain, the carboxy-terminal association domain and a variable region that can undergo alternative splicing to generate a variety of CaMKII subtypes. Several posttranslational modifications in the regulatory domain positively and negatively modulate CaMKII activity. **B** CaMKII holoenzyme structure, consisting of 12 subunits. **C** Automatic phosphorylation of CaMKII requires the participation of T286 site

Calcium/calmodulin-dependent protein kinase II (CaMKII) is a family of serine (Ser)/threonine (Thr) protein kinases with extensive substrate specificity. It is ubiquitously expressed in biologic tissue and is able to regulate various calcium-modulated functional responses relying on cellular microenvironment. The CaMKII family has four genes: *Camk2a*, *Camk2b*, *Camk2g*, and *Camk2d*, which encode four isoforms: CaMKIIα, CaMKIIβ, CaMKIIγ, and CaMKIIδ respectively. It is a common occurrence for tissue to express more than one isoform of CaMKII. All the four genes of CaMKII family have a highly conserved architecture, which consists of a kinase domain (exons 1–10), a regulatory region (exons 11–12), a variable linker segment (exons 13–21), and a hub domain (exons 22–24). In this context, the structures of kinase encoded by these four genes are roughly the same, and each subunit consists of three conserved domains: the amino-terminal catalytic domain, the central self-regulating domain and the carboxy-terminal association domain (Fig. 1A). The catalytic domain contains ATP and substrate binding bags, which provide the catalytic activity of proteins [10]. The self-regulating domain consists of an inhibitory pseudosubstrate sequence, several post-translational modification sites and calmodulin binding regions. These two domains are closely correlated with the CaMKII phosphorylation [11]. The association domain is responsible for the oligomerization of subunits to form holoenzymes. Actually, CaMKII necessitates the holoenzyme structure for exerting its regulatory functions, such as the autophosphorylation at T286 [12]. The CaMKII holoenzyme is gathered by the association of 12 or 14 subunits through their C-terminal association domains (also known as hub domains) (Fig. 1B). Its subunit composition could be an assembly of identical isoforms (homomeric) or a combination of different isoforms (heteromeric), conferring unique properties to CaMKII [13]. Upon activation, the CaMKII holoenzyme enables CaMKII subunits to exchange between different holoenzymes, thereby inducing the spread of activation state in the process, which would possibly be essential for the storage of long-term information in the brain [14]. Moreover, the association domain contains a linker with variable length, which can produce around 40 different subtypes for the four isoforms by selective splicing. Additional splice sites are also found in the genes of CaMKII on linker segment, and they will facilitate the sub-cellular localization. For example, CaMKIIβ and γ are able to localize to F-actin as long as exon 13 is spliced in [15]. Moreover, selective splicing taking place in the linker influences the phosphorylation of CaMKII. Previous researches have shown that CaMKIIα (30-residue linker) is more readily phosphorylated at Thr286 than at Thr305/306, while the opposite is true for CaMKIIβ (a 217-residue linker). These data suggest that linker length/sequence may also affect the balance between activation and inhibition of CaMKII. A detailed review of CaMKII alternative splicing is provided by Sloutsky and Stratton [16].

## Different roles of CaMKII isoforms in the retina

The isoforms of the CaMKII family are similar in terms of structure but different in function and distribution. In the retina, the expression of CaMKIIα and -β is restricted to ganglion and amacrine cells [17, 18], whereas CaMKIIγ and -δ are diffusely distributed and are the major isoforms expressed in endothelial cells [19], suggesting that isoform-specific distribution is relatively tissue selective. In this context, CaMKII isoforms play different roles in distinct cells where they are expressed. For instance, CaMKII acts as a critical mediator of retinal angiogenesis both in vitro and in vivo [20]. Specifically, in human umbilical vein endothelial cells, CaMKIIδ upregulation is facilitated by the pro-inflammatory cytokine interleukin 6 (IL-6) through a STAT3 (signal transducer and activator of transcription factor 3)-dependent mechanism, thereby promoting the endothelial motility, proliferation, and in vivo angiogenesis [21]. Moreover, CaMKIIδ is required for DR, a complication of diabetes characterized by neovascularization. However, different CaMKII isoforms regulate disparate pathways and exert contrasting effects on the angiogenic action. CaMKIIγ and -δ are involved in the growth factor-induced retinal angiogenesis in vitro, but they are implicated in different signaling pathways: CaMKIIγ is more responsive to the insulin-like growth factor-1, whereas CaMKIIδ is preferentially activated by vascular endothelial growth factor (VEGF) and basic fibroblast growth factor [19]. Moreover, genetic deletion of CaMKIIγ and -δ elicits distinctive outcomes in the oxygen-induced retinopathy (OIR) model. Specifically, in the OIR model, CaMKIIγ-KO mice enhance reparative angiogenesis in the ischemic tissue, while CaMKIIδ-KO retina may induce inhibitory effects on this process [19]. The disparate effects may be ascribed to the different distributions of isoforms. Simultaneously, the spatial and temporal variations of CaMKII expression in the whole-mount of zebrafish consistent with pleiotropic roles during retinal development [22]. However, further explorations are need to recognize the upstream regulators or downstream substrates of CaMKIIγ and -δ, thereby identifying the specific pathways in which they function.

In electrical synapses, CaMKII plays an integral role in modulating the coupling strengths. However, CaMKIIα is strongly expressed in starburst amacrine cells, where electrical coupling is known to be absent [17]. Consequently, modulation of electrical synapses is not the sole role fulfilled by CaMKIIα in these cells. It is now recognized that CaMKIIα may play a role in regulating the neurotransmitter release of starburst amacrine cells [23]. Besides, CaMKIIγ is diffusely distributed throughout the retina and hence it cannot be assigned to a specific cell type [24]. Nonetheless, there are differences in the composition of enzymes colocalizes with the electrical synapses. In concrete terms, only CaMKIIβ colocalizes with gap junctions in the outer retina, whereas both CaMKIIβ and CaMKIIδ colocalize with gap junctions in the inner retina [24]. This localization may have a potentially physiological significance for the modulation of electrical synapses. Furthermore, several researches have described the discrepancy in the binding affinity and autophosphorylation of CaMKII isoforms [25]. As a consequence, the different distributions of CaMKII isoforms may reflect the unique properties of the electrical synapse networks they mediate. Notably, a certain isoform of CaMKII can exert unique protective effect on retinal ganglion cells (RGCs). It was demonstrated that levels of CaMKIIαB, a nuclear isoform of CaMKIIα, increase significantly during the NMDA-induced retinal degeneration [26]. CaMKIIα, one of the dominant isoforms in the retina, is exclusively distributed in the ganglion and amacrine cells. CaMKIIαB derives from the alternative splicing of α gene and contains a nuclear localizing signal, facilitating the translocation of CaMKIIα to the nucleus. Different localizations of CaMKIIα lead to contrasting results: cytoplasmically localized CaMKIIα is involved in mediating RGCs death through suppressing the release of brain-derived neurotrophic factor (BDNF) [27], whereas the nuclear localized CaMKIIα (and CaMKIIαB) in essential for cell survival [26]. When the retina is exposed to the NMDA mediate cytotoxic effects, the activity of CaMKIIα together with CaMKIIαB alter profoundly [28]. BDNF is a neurotrophin that produced by neuroglial cells in the retina. It has been demonstrated experimentally that knockdown of CaMKIIα gene by siRNA can reduce the BDNF expression and accelerate the RGCs death [26]. In addition, it has been reported that B-cell lymphoma-2 (Bcl-2), a well-known antiapoptotic gene, is also suppressed by the knockdown of CaMKIIα [29]. Conversely, overexpression of CaMKIIα upregulated Bcl-2 expression, in an in vitro RGCs model that is exposed to glutamate toxicity [26]. Nonetheless, an independent study shows that CaMKII-α blockade effectively hindered NMDA-induced caspase-3 activation, providing substantial neuroprotection against the NMDA-evoked RGCs loss in a rat retinal toxicity model [30]. Currently, there is a lack of clinical trials investigating the role of CaMKII and its subtypes in retinal health. A review of existing literature revealed limited clinical trials focusing on CaMKII (Table 1).

**Table 1:** Summary of clinical trials targeting CaMKII

Target	Experimental objects	Treatment	Method	Results	Ref
CaMKII	113 patients undergoing elective coronary artery bypass grafting	AIP, KN93	Right atrial appendage biopsies, western blotting	Patients with sleep-disordered breathing show increased atrial CaMKII activity resulting in proarrhythmic Na current dysregulation	[31]
CaMKIIδD	Twenty-four young and healthy men	8-week velocity-based resistance training program	Muscle biopsies, protein extraction, western blotting, MHC composition	The changes in phospho-Thr287-CaMKIIδD were positively associated with muscle hypertrophy and the number of repetitions during training	[32]
CaMKII	Human synovial fibroblasts isolated obtained from knee replacement surgeries of 18 patients with OA	Adiponectin, AMPK inhibitors (AraA and compound C)	Flow cytometry analysis, transfection of siRNAs, cell adhesion assay	Adiponectin increases ICAM-1 expression in human OASFs via the LKB1/CaMKII, AMPK, c-Jun, and AP-1 signaling pathway	[33]
CaMKII/p-CaMKII	Rats received saline + placebo, ISO + placebo, or ISO + dapagliflozin	Dapagliflozin	Ca^2+^ imaging study in LVCMs, measurement of cell contraction, echocardiographic analysis	Dapagliflozin treatment reduced the expression of CaMKII and p-CaMKII in ventricles from isoproterenol-treated rats	[34]
CaMKII	20 participants with acute spinal cord injury	Comb-NMES training	Enzymatic assays, western blots	Comb-NMES training can upregulate CaMKII in skeletal muscle of patients with spinal cord injury	[35]
CaMKIIα	18 patients with sickle cell disease	Trifluoperazine	Visual analogue scale for pain intensity, extrapyramidal symptom rating scale	Trifluoperazine shows promise as an analgesic drug in adults with sickle cell disease	[36]
CaMKII	13 recreationally active male subjects with a weekly training	Formoterol	Muscle analysis, blood analysis,	The long-acting 2-agonist formoterol increased quadriceps muscle strength and power output during 30 s of maximal sprinting	[37]
CaMKII	Slice patches made from CA1 pyramidal neurons in rat hippocampal slices	Slice patches made from CA1 pyramidal neurons in rat hippocampal slices	Slice patches made from CA1 pyramidal neurons in rat hippocampal slices	Slice patches made from CA1 pyramidal neurons in rat hippocampal slices	[38]

Abbreviations: *ICAM-1* intercell adhesion molecule-1, *OA* osteoarthritis, *LKB1* liver kinase B1, *OASFs* OA synovial fibroblasts, *AMPK* AMP-activated protein kinase, *ISO* isoproterenol, *LVCMs* left ventricular cardiomyocytes, *Comb-NMES* (aerobic + resistance) neuromuscular electrical stimulation

## Metabolism and regulatory mechanisms of CaMKII

Under physiological conditions, each subunit of CaMKII is kept in a basal/inactive state through the interactions between its catalytic domain and the autoinhibitory region within the regulatory domain [39]. When the cell is exposed to pathological stimuli, CaMKII will be activated by upstream signals, including calcium ions, angiotensin II, aldosterone, nitric oxide, and hyperglycemia. These upstream regulators can affect the CaMKII activity via activating or inhibiting posttranslational modifications (PTMs) [40]. Figure 1A shows some critical regulatory sites of CaMKII, including those involved in phosphorylation, oxidation, O-GlcNAcylation and S-nitrosylation.

### Phosphorylation of CaMKII

CaMKII activation can regulate downstream signaling through phosphorylation. The alpha helical structure of the autoinhibitory region of CaMKII consists of two sequences that overlap with each other, namely an auto-inhibition sequence and a CaM binding sequence. The conserved threonine 286 (T286) (T287 for CaMKIIβ, δ,γ) phosphorylation site resides in the autoinhibition sequence. T286 amino acid binds to the conservative position in the catalytic domain to maintain the inactive conformation [39]. When the intracellular Ca^2+^ level increases, Ca^2+^ binds CaM to form the Ca^2+^/CaM complex. This complex combines to the binding site in the regulatory domain to induce a conformational change that allows the catalytic domain to be dissociated from the autoinhibitory region. This will expose the substrate binding sites in the catalytic domain, indicating the transient activation of CaMKII. However, the transient activation is reversible: the decrease in calcium ions allows CaM to decalcify and dissociate from CaMKII, so that the pseudosubstrate re-inhibits kinase activity [40]. Individual subunits of CaMKII are activated separately in the holoenzyme. The conformational change caused by sustained Ca^2+^/CaM binding also exposes the conservative phosphorylation site of T286 in the self-inhibitory region, so that it could be phosphorylated by adjacent activated subunits [39]. This phosphorylation event increases the affinity for CaM by 1000 times and prevents the recombination of catalytic domains, thus maintaining enzyme activity even in the absence of Ca^2+^/ CaM.

In different molecular environments, dephosphorylated CaMKII combines with diverse proteins. A stimulus-induced rise in intracellular calcium activates CaMKII, subsequently causing different patterns of phosphorylation of both/either CaMKII themselves and/or binding proteins. This makes CaMKII dissociate from the proteins and then integrate with other specific binding proteins, which can connect with various molecular pathways and translocate the CaMKII to another intracellular position, thereby incurring different functional outcomes after the initial CaMKII activation [41]. In this context, several phosphorylation sites have no direct impact on CaMKII activity but deeply affects the targeting specificity of CaMKII. For instance, the phosphorylation of T253 has no direct effect on the kinase activity or Ca^2+^/CaM binding to CaMKII but affects the targeting specificity of CaMKII via interactions with other binding proteins, thereby exerting effects on cell physiology [39], probably related to metaphase–anaphase transition, ischemia- or excitotoxicity-induced cell death, postsynaptic density and so on. Phosphorylation at each of all these sites can also alter the binding of CaMKII to specific proteins and then change the functional responses of CaMKII.

CaMKII inactivation could be mediated through underlying mechanisms: the phosphatase-dependent and phosphatase-independent pathway, respectively. Protein phosphatase (PP) is a kind of Ser/Thr phosphatases that dephosphorylates cellular phosphoproteins, regulating CaMKII via dephosphorylating both CaMKII and its binding proteins [41]. Dephosphorylation of Thr286 occurs through 70% protein phosphatase 2A (PP2A) activity, with PP1 and PP2C responsible for the remaining 30% [42]. The other CaMKII inactivation mechanism is typical of post-synaptic plasticity regulation. Autophosphorylation of Thr305/306 can modify the Ca^2+^/CaM binding site, consequently impeding the reattachment of CaM to the regulatory domain [43].

### Oxidation of CaMKII

Reactive oxygen species (ROS), which is a cluster of highly reactive chemical species containing oxygen, can be produced during the redox reactions. These physiologically produced ROS are constantly cleared by antioxidant systems. Furthermore, slight ROS-induced damages can also be repaired and compensated to maintain the dynamic balance of redox biology in the body. Oxidative stress is caused by excessive production of ROS, which can damage the structural lipids or proteins, and contribute to the pathogenesis of RD. CaMKII expression has been shown to be affected by oxidation, which may occur in subcellular domains such as mitochondria [44]. ROS generated from multiple sources, including NADPH oxidase, malfunctioning mitochondria and abnormal neurohormonal signaling, can lead to oxidation of CaMKII [44]. For example, in the AC3-I transgenic mouse model of cardiac CaMKII inhibition, aldosterone treatment leads to excessive ROS production, and the subsequent oxidation of CaMKII [45]. As CaMKII is activated by Ca^2+^, the self-inhibitory domain of CaMKII will be removed from the catalytic domain. ROS can induce the oxidative modification of the conserved methionine pair M281/282 (C281/M282 in CaMKIIα) in the CaMKII self-inhibition region, thereby generating an oxidized form of CaMKII [46]. Oxidized methionine prevents the reassociation of the autoinhibitory region with the catalytic domain following Ca^2+^/CaM dissociation, resulting in an autonomously active state similar to T286 autophosphorylation. Molecules interacting with methionine monooxygenase CASL1 and methionine sulfoxide reductase B can dynamically regulate the redox state on M308 [47]. On the other hand, the oxidation of CaMKII has been shown to be a reversible event, which can be reversed by intracellular methionine sulfoxide reductase [48]. Furthermore, treating diabetic mice with mitochondrion-targeted antioxidants can reduce the level of oxidation CaMKII (ox-CaMKII) and alleviate the cardiomyocyte death in mouse hearts [49].

The increased level of ox-CaMKII is also closely correlated with inflammatory response. Excessive inflammation further integrates ROS production, indicating that there exists a positive feedback loop between oxidative stress and inflammation [40]. In a CaMKII-deleted mouse model, the expression of inflammatory genes is down regulated compared with normal control mice after I/R injury [50]. Studies also show that ox-CaMKII can enhance pro-inflammatory transcriptional signaling through enhancing the activity of NF-κB [51]. Therefore, CaMKII seems to be a node signal, linking the elevated ROS “upstream” signals with “downstream” inflammatory events through the action on multiple targets. Activating kinases, requires that the enzyme that is initially “turned on” by Ca^2+^/CaM to allow access to the self-inhibitory domain for oxidation or self-phosphorylation, and to prevent the interaction between the self-inhibitory domain and the catalytic domain after activation, thus providing continuous Ca^2+^-independent CaMKII activation [44]. CaMKII activated by ROS can also regulate redox regulatory factors such as NADPH and reduced glutathione, and further aggravate the oxidative damage. Targeted removal of ROS can lead to the reduced level of CaMKII [48, 49]. In essence, the oxidation of the methionine residue in CaMKII acts as a sensor for ROS increment, and is related to the persistent kinase activity [48, 49].

### Protein–protein interaction

CaMKII substrates are selectively distributed in different subcellular locations, and the protein–protein interaction can promote the recruitment of CaMKII to these sites [39]. The cellular membrane is the main site for interaction between CaMKII and proteins. In the neurons, glutamate can activate the postsynaptic NMDA receptor subunit GluN2B, which binds and recruits CaMKII to the synapse [52]. In addition, CaMKII has a broad spectrum of other protein binding partners: connexin36 binds with CaMKII to recruit it to gap junctions [53], and the α subunit of L-type Ca^2+^ channels can interact with CaMKII to mediate the Ca^2+^-dependent facilitation of voltage-gated calcium current [54]. Besides membrane association, CaMKII can also be mobilized to other subcellular locations such as the cytoskeleton through binding to F-actin [55].

### Noncoding RNAs

Noncoding RNA (ncRNA) is a kind of RNA that has versatile biological function but does not encode protein. They can regulate the expression of target genes at epigenetic, transcriptional, and post-transcriptional levels [39]. There are two main types of ncRNAs, one is short RNA with a length of less than 200 nucleotides, and the other is long ncRNA (lncRNA) with a length of more than 200 nucleotides. Several lines of evidences suggest that they can regulate the expression of CaMKII gene to some extent. For example, microRNA145 can downregulate the CaMKIIδ expression in cardiomyocytes and reduce Ca^2+^ overload [56]. Moreover, terminal differentiation induced ncRNA, a 3.7-kb lncRNA, can attenuate myocardial hypertrophy through epigenetically silencing of CaMKII [57]. *Camk2d*-associated transcripts 1, the first CaMKII regulatory lncRNA identified thus far, can promote neuronal survival in a focal cerebral ischemia and reperfusion mouse model through the NF-κB signaling pathway [58]. *Camk2d*-associated transcripts 2 can regulate the CaMKIIδ expression in the neurons [58]. A recent study identified a previously uncharacterized lncRNA *Carip* can specifically interact with CaMKIIβ, thereby modulating long-term synaptic plasticity and affect spatial learning function of mice [59]. However, the regulatory mechanism of ncRNA in retina has not been fully elucidated.

## The role of CaMKII in the eye

### The mechanism of CaMKII promoting angiogenesis

**Fig. 2:**
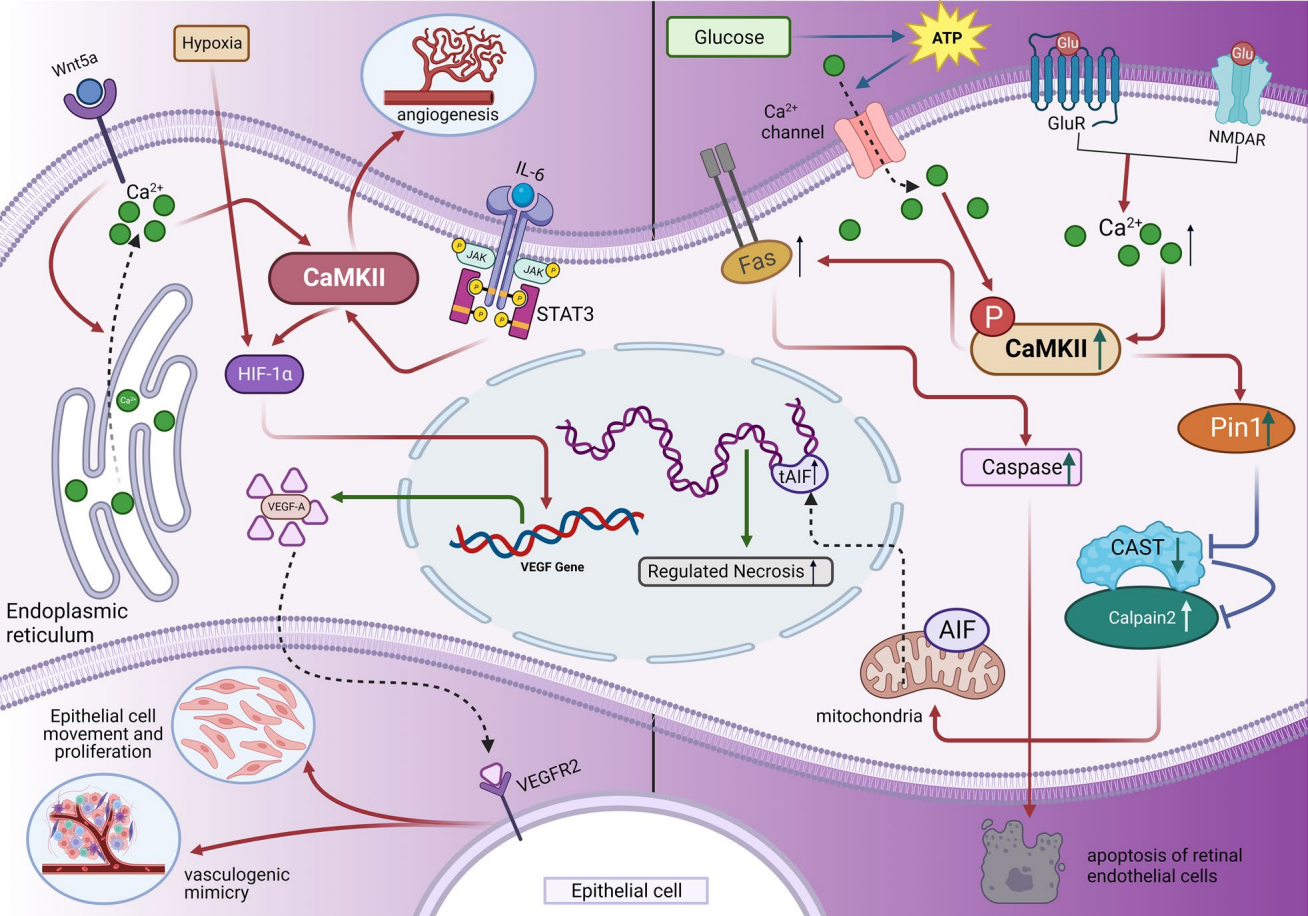
The CaMKII signaling pathway promotes angiogenesis in the retina. The mechanism by which CaMKII leads to apoptosis and regulates necrosis is through calcium overload. The red arrows denote activating signals, while the blue arrows indicate inhibitory signals. The green arrows denote transcription and translation, while the dotted lines indicate the movement of the position

Angiogenesis is the process in which new capillaries are formed from existing vessels, and serves as an essential step for normal retinal development and wound healing [60]. However, abnormal angiogenesis is a pathological hallmark of numerous eye diseases, such as retinopathy of prematurity (ROP), DR, and AMD [61, 62]. VEGF, a hypoxia-inducible growth factor, plays a key role in the development of pathologic angiogenesis [63]. Typically, VEGF exerts angiogenic effects through the binding to its receptor, VEGFR2. Previous studies have demonstrated that VEGF-evoked activation of VEGFR2 induces phospholipase C (PLC)-dependent production of inositol 1,4,5-trisphosphate (IP_3_) [64]. IP_3_ leads to Ca^2+^ release from the endoplasmic reticulum via acting on its specific receptor, which subsequently activates the CaMKII [20]. Therefore, the VEGF-induced retinal angiogenesis is dependent on CaMKII activation. The downstream pathways of CaMKII remain enigmatic. It has been shown that phosphatidylinositol-3 kinase (PI3K)/Akt signaling is intimately associated with the activation of CaMKII and its upstream PLC/IP_3_/Ca^2+^ pathway [20, 65]. PI3K/Akt signaling axis is critical for multiple steps of angiogenesis, including endothelial cell migration, reproduction, and vessel formation [66]. It is demonstrated that VEGF-modulated Akt phosphorylation in retinal endothelial cells demands the activation of both CaMKII and the PI3K pathways in that the blockade of either abrogates Akt activation [20, 67]. Therefore, CaMKII may act as a therapeutic target for the inhibiting the angiogenesis (Fig. 2).

STAT3 severs as a key transcription factor to modulating the pathological process of retinal angiogenesis [68]. IL-6, a pro-inflammatory cytokine in endothelial cells, plays a vital role in retinal inflammation and pathological neovascularization. Actually, IL-6 has the capacity of activating STAT3 via the janus kinase [69]. The suppressor of cytokine signaling 3 (SOCS3) is the predominant downstream target of STAT3. However, SOCS3 can suppress STAT3 activation in turn via a negative feedback mechanism and functions as a negative regulator of retinal vascularization [70]. Moreover, CaMKII also acts as a potential downstream target of STAT3 during the process of angiogenesis. In contrast to SOCS3, CaMKII facilitates the process of retinal angiogenesis. As aforementioned, CaMKIIδ and CaMKIIγ are the major isoforms of CaMKII expressed in endothelial cells [19]. Emerging evidence suggests that both IL-6 and STAT3 induce an increase in the expression of CaMKIIδ in human umbilical vein endothelial cells [21]. Consequently, it is reasonable that IL-6 is able to upregulate the STAT3-dependent CaMKIIδ expression, thereby promoting the endothelial cell migration, proliferation, as well as angiogenesis [21]. Even though the upstream activation signals of CaMKIIδ have been clarified, the downstream targets of CaMKII still remain enigmatic. Accordingly, it is crucial to verify exactly how CaMKII, as a kinase with extensive substrate specificity, acts on target molecules and fulfills a range of functions that belong to endothelial cells.

Previous studies have described the potential relationship between Wnt5a/CaMKII signaling pathway and vasculogenic mimicry (VM) [71]. VM is a tumor blood supply system that takes place independently of angiogenesis or endothelial cells, forming vascular structures in tumor tissue [72]. In non-canonical Wnt5a/Ca^2+^ pathway, Wnt5a binds to its specific receptor Frizzled and evokes the release of Ca^2+^ ions from intracellular stores, which subsequently activates CaMKII [73]. CaMKII is considered as a critical regulator of retinal neovascularization induced by diverse growth factors [19]. Additionally, although the specific signaling pathways remain unclear, the Wnt5a/CaMKII pathway is proven to be involved in upregulating the expression of molecules related to VM formation and angiogenesis, including the HIF-1α, VEGFR2, VEGFA, vascular endothelial (VE)-cadherin, and ephrin type-A receptor 2 (EphA2) [71]. Hypoxia-inducible factors are a group of transcriptional activators, serving as the primary regulators of hypoxia-modulated angiogenic stimulators. Under hypoxic conditions, the HIF-1α expression in nuclear increases, and promotes the transcription of VEGFA. VEGFA is able to modulate the proliferation, migration and survival of vascular endothelial cells through activating VEGFR2, a specific VEGFA receptor on endothelial cells. Besides its critical contribution to VM formation, VEGFR2 also plays an important role in promoting endothelial cell mitogenesis and retinal vascular permeability [74]. Additionally, VE-cadherin and EphA2 are two other proteins identified as having a role in mediating VM formation. Studies have shown that downregulation of VE-cadherin or EphA2 inhibits VM formation [75]. In this context, the Wnt5a/CaMKII pathway may act as a potential therapeutic target for suppressing the growth of intraocular malignant tumors.

### Dual effects of CaMKII on retinal pigment epithelium

CaMKII can prevent the retinal pigment epithelium (RPE) from oxidative stress and apoptosis through ameliorating the mitochondrial dysfunction [76]. Once the retinal cells are injured by pathological insults, overloaded calcium activates diverse calcium-dependent enzymes. Among them, the activated CaMKII can phosphorylate the adenosine 5‘-monophosphate-activated protein kinase (AMPK) and induce the AMPK activation [76]. It has been shown that KN93, a CaMKII inhibitor, can suppress the AMPK activation [77]. Previous research has shown that Wnt inhibitory factor 1, an antagonist of Wnt/β-catenin signaling, can alleviate mitochondrial dysfunction in ARPE-19 cells pre-conditioned with high glucose [78]. However, AMPK alleviates oxidative stress via inhibiting the activation of NADPH oxidase, which can promote the production of intracellular ROS [79]. Moreover, CaMKII-mediated AMPK activation can also ameliorate the ROS-triggered mitochondrial dysfunction [76, 80]. During this process, these cytosis ROS promotes the open of mitochondrial permeability transition pore and the subsequent release of cytochrome c, giving rise to a caspase cascade-regulated RPE apoptosis [81]. However, the CaMKII-mediated protective effects would be reversed due to AMPK knockdown.

Notably, accumulating evidence suggests that CaMKII can also contribute to the RPE cell loss under specific conditions. When the retina is exposed to excessive blue light, ROS overproduction will activate CaMKII, which subsequently phosphorylates the dynamin-related protein 1 (Drp1) at Ser616 and promotes mitochondrial fission [82]. The balance between mitochondrial fission and fusion is extremely essential for maintaining the normal function of RPE cells [83]. As a consequence, the disruption to this equilibrium can result in mitochondrial dysfunction and subsequent overproduction of ROS, ultimately forming a vicious cycle to induce RPE apoptosis. Although the full signaling pathway remains elusive, it has been reported that CaMKII-mediated Drp1 phosphorylation is closely related to the apoptosis-inducing factor (AIF) activation under blue light exposure [82]. As a mitochondrial inner membrane protein, AIF can be cleaved and translocated from mitochondria to nucleus and then cause RPE cell apoptosis or necroptosis in a caspase-independent manner [84]. The specific regulatory relationship among the three proteins still requires deeper exploration. Therefore, CaMKII can be a therapeutic target in retinal degenerative diseases, which are characterized by aberrant function and/or eventual death of RPE cells. Nevertheless, given its protective and disruptive dual functions, researchers ought to clearly determine how to guide CaMKII to phosphorylate appropriate substrate, thereby activating proper signaling pathways and ultimately achieving the desired therapeutic outcomes.

### CaMKII and calcium overload

Calcium overload is a pathological event that characterized by the abnormally high level of intracellular calcium and the disturbed metabolism. The activation of CaMKII, a protein kinase dependent on calcium and calmodulin, is intricately linked to the calcium levels within the body [85]. A growing body of evidences show that calcium overload can induce the over-phosphorylation of CaMKII, and elicit detrimental effects on cardiomyocytes, neurons [86]. In the retina, the calcium overload-induced cell injury has also been found to be correlated with the CaMKII activation [87]. For instance, glutamate is a ubiquitous excitatory neurotransmitter that can modulate the physiological activity of retinal neurons via activating the glutamate receptors (GluRs) [88]. However, excessive glutamate can induce the overstimulation of GluRs in the retina, and promote excessive calcium influx, which ultimately leads to the death of retinal neurons [89]. Specifically, the calcium overload-induced CaMKII activation can upregulate the peptidyl-prolyl isomerase 1 (Pin1) expression in primary cultures of retinal neurons prepared from 1-day-old rat pups [87]. Pin1 is a subtype of the peptidyl cis-to-trans isomerases, which can bind and catalyze the cis/trans isomerization of phosphorylated threonine/serine–proline.

Notably, the Pin1 protein can suppress the activation of the downstream molecule calpastatin (CAST), a specific endogenous inhibitor of calpain2 [90]. Through this mechanism, Pin1 indirectly enhances the activity of calpain2, a prototypical isoform of the calcium-dependent cysteine proteases. Researchers have detected the simultaneously upregulated levels of truncated AIF protein and calpain2 in glutamate-induced retinal neuronal regulated necrosis (RN) [90]. Eventually, the calpain-cleaved AIF translocate to the nucleus, whereby initiating the DNA degradation and RN [91]. These findings enrich our understandings of the mechanism underlying the CaMKII mediated detrimental effects on retina. Further investigation is required to explore the exact relationship among calcium overload, CaMKII, and retinal lesion through additional experimental studies. Thus far, mounting experiments have yielded evidences that calcium channel blockers (CCBs), such as diltiazem, nicardipine, verapamil, and nifedipine, have the capacity to mitigate renal failure, cardiomyocyte apoptosis, mitochondrial fragmentation, and retinal dysfunction through inhibiting the calcium overloading [92, 93]. However, the precise signaling pathway by which CCBs affect CaMKII in the retina remains enigmatic.

### CaMKII regulates retinal ribbon synapses

In the retina, the ribbon synapses which contain a specific membrane-attached structure, are found almost exclusively in the outer plexiform layer [94]. The synaptic ribbons are presynaptic protein complexes which act as important mediators for the transmission of sensory information. The neurotransmitters release via the exocytosis of synaptic vesicles is believed to be the pivotal process of visual signaling transmission [95]. SNARE (soluble N-ethylmaleimide-sensitive factor attachment protein receptor) is an extremely conserved family of membrane-associated proteins that can regulate the intracellular membrane fusion. The vesicle fusion, an essential step for release of neurotransmitters, requires the interaction between v-SNARE localized on synaptic vesicles and t-SNARE localized on synaptic ribbons [96]. The t-SNARE consists of synaptosomal-associated protein 25 kDa (SNAP-25) together with syntaxin 3B, a protein that attached to the presynaptic membrane. Syntaxin 3B, requires a shift from closed to open conform process the capacity of binding SNAP-25 and subsequently engaging in neurotransmitters release [97]. In contrast to the related isoform syntaxin 1A expressed in conventional synapses, syntaxin 3B bounds SNAP-25 with a lower affinity and thus is inefficient to catalyze the vesicle fusion [98]. Therefore, mutations in ribbon synapses will induce defect in sensory transmission and cause visual impairments. Emerging evidences suggest that CaMKII-mediated syntaxin 3B phosphorylation can modulate the exocytosis of synaptic vesicles [4]. T14 site in N-terminal domain of syntaxin 3B is a substrate of CaMKII [99]. The phosphorylated site via CaMKII may switch syntaxin 3B into a more open conformation like the configuration change of syntaxin 1A [99]. As aforementioned, although syntaxin 3B is initially weakly attached to SNAP-25, the later open conformation caused by phosphorylation can react with SNAP-25 to form a more stable t-SNARE complex that facilitates neurotransmitter release. Researchers hypothesize that the phosphorylation-modulated syntaxin 3B affinity with SNAP-25 is a unique regulatory mechanism in response to graded changes [99]. Rod photoreceptors can detect single photons but are nevertheless able to sense the changes in light intensity within a range of 10 to 10^−6^ cd/m^2^ [100]. In order to adapt to the ambient environment, the retina needs to transmit graded information with high fidelity across a broad range of stimulus intensity and for a long period of time, which places stringent demands on its release machinery. A trial has shown that phosphorylation of syntaxin 3B is regulated by light stimulus via a Ca^2+^-dependent pathway [4]. The researchers find that both dark-adapted rod photoreceptors and light-exposed rod bipolar cells in mouse retina exhibit upregulated levels of phosphorylated syntaxin 3B [4]. Calcium influx through voltage-gated calcium channels occurs in the terminals of bipolar cells and rod photoreceptors upon depolarization [101]. Subsequently, activated CaMKII leads to the phosphorylation of syntaxin 3B, and triggering the release of neurotransmitters. However, the ratio of phosphorylated syntaxin 3B in cone photoreceptors did not demonstrate any significant response to the alteration in light exposure [4]. This phenomenon can be attributed to the different regulation mechanism in rods and cone photoreceptors. Rods demonstrate graded regulation in response to varying levels of illumination, while cones necessitate the ability to maintain synaptically-functional under photopic conditions [102]. To date a substantial amount of literatures suggest that CaMKII plays a significant role in various physiological processes associated with syntaxin 1A. These processes include the facilitation of dopamine efflux in brain and the modulation of serotonin transporters [103]. However, the exact relationship between CaMKII and syntaxin 3B remains elusive. Further study is required to investigate the correlation between CaMKII and syntaxin 3B in retinal pathology or physiology.

### Cellular and molecular mechanisms of CaMKII influencing retinal degeneration

**Fig. 3:**
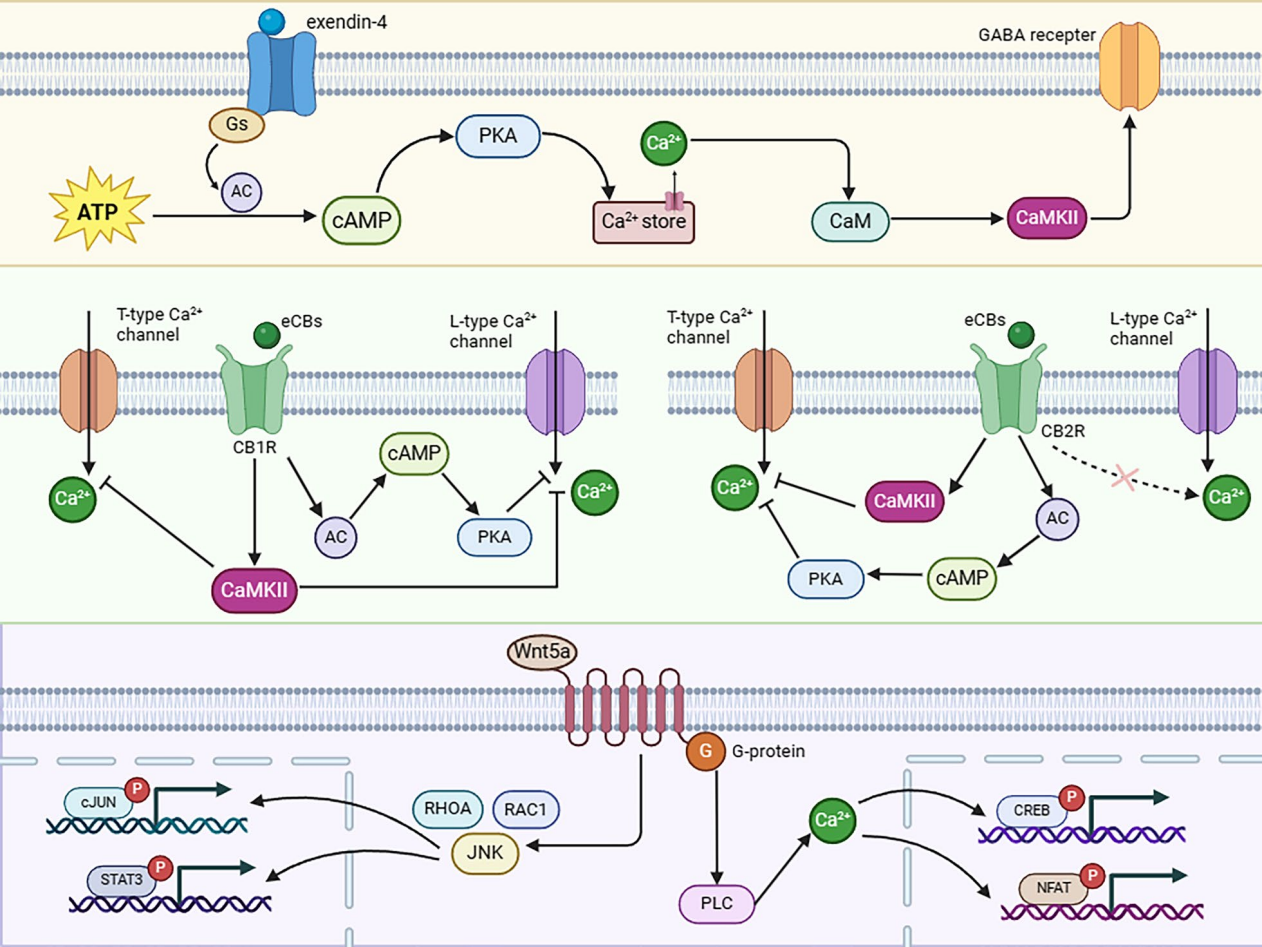
Cellular and molecular pathways of CaMKII influencing retinal degeneration. Abbreviations: AC, adenylate cyclase; eCBs, endogenous cannabinoids; CB1R, cannabinoid CB1 receptors; CB2R, cannabinoid CB1 receptors; RHOA, Ras homolog gene family member A; JNK, c-Jun N-terminal kinase; RAC1, Rac family small GTPase 1

Retinal degeneration, a leading cause of vision impairment globally, is marked by the demise of various retinal cells. The pathological process of retinal degeneration is complex, with CaMKII signaling playing a role potentially (Fig. 3). For instance, DR is a type of retinal degeneration, with abnormal neovascularization. CaMKII is implicated in the growth of abnormal angiogenesis through the activating the PI3K/Akt signaling axis. Retinal degeneration is frequently accompanied by retinal remodeling, ultimately leading to death of retinal neurons in the inner layers. The progressive loss of RGCs is typically irreversible. Protein kinase A (PKA) and CaMKII signaling pathways are known to modulate the excitability of these cells through targeting the dopamine D1 receptor on RGCs [104]. In rat retinal slices, the activation of D1 receptors by dopamine regulates the firing of RGCs through modulating hyperpolarization-activated cationic currents. A study has shown that D1 receptor activation in rat RGCs inhibits the outward potassium currents through intracellular signaling pathways involving PKA and CaMKII [104]. Furthermore, the binding of endogenous cannabinoids and their ligands can also activate PKA and CaMKII signaling pathways, leading to the inhibition of L- and T-type calcium channels and ultimately regulating the excitability of RGCs [105]. In addition, the GABA receptor on the RGCs mediates the inhibitory signals from non-secretory cells. The PKA/CaMKII signaling pathways is shown to facilitate the inhibitory impact of agonist exendin-4 on GABA receptors [106]. Furthermore, Wnt5a signaling can enhance the retinal axon growth and provide protection for RGCs. Wnt5a binds to its receptor, initiating a signaling cascade involving the Rac family small GTPase 1, Ras homolog gene family member A, and c-Jun N-terminal kinase (JNK). Activation of the Wnt/Ca^2+^ signal triggers the activation of heterotrimeric G proteins, leading to phospholipase C activation and the release of intracellular calcium stores [107]. Wnt5a has been shown to activate phosphorylation of CaMKII and JNK, leading to the upregulation of downstream pathway components. Given its role in promoting the growth of adult retinal axons, Wnt5a represents a potential target for protecting the RGCs.

## CaMKII and eye diseases

### Glaucomatic optic neuropathy

Glaucomatic optic neuropathy is characterized by the progressive degeneration of RGCs and abnormal changes in the optic nerve head [108]. It is widely recognized as a prominent cause of global blindness that impacts a staggering population of over 70 million individuals [109]. This disease is distinguished by the gradual deterioration of RGCs, resulting in the progressive optic disc atrophy and visual impairment [108]. Although the elevated intraocular pressure is presently recognized as the primary risk factor for the initiation and progression of glaucoma, additional pathogenesis factors, including oxidative stress, immune factors, genetic predisposition, and lifestyle choices, may also exert influence [110]. Elevated intraocular pressure in individuals with glaucoma can cause mechanical axonal damage through impeding the retrograde transfer of vital nutritional factors from brain stem cells to RGCs [111]. Given the limited regenerative capacity of RGCs, the degeneration of RGCs axons will cause permanent vision impairment. Consequently, it is crucial for glaucoma patients to safeguard their RGCs and axons against various forms of pathological insults.

The activation of exchange proteins activated by cAMP (Epac) 1 can cause RGCs death via activating CaMKII [112]. Epac is a novel cAMP mediator that comprises two subtypes, namely Epac1 and Epac2. The Epac1 is expressed in the retinal layer housing neurons, while Epac2 is expressed mainly in neuroendocrine and endocrine tissue [113]. Epac1 possesses the ability to induce RGCs death. On the one hand, intraperitoneal injection of ESI-09, a potent inhibitor of Epac, can partially inhibit the Epac activity after ischemic injury and reduce the RGCs loss in I/R model [112]. Notably, there exists a close correlation between Epac1 activation and CaMKII activity. In the glaucoma mouse model, the expressions of Epac1 and its upstream activator cAMP increase prominently, accompanied by concurrent up-regulation of CaMKII phosphorylation. To corroborate these findings, depletion of Epac1 can inhibit CaMKII activation in mouse retinas [112]. Emerging evidences suggest that the RGCs death in glaucomatous retina might be caused by the Epac1-induced CaMKII-activation. CaMKII is promptly activated in an Epac1-dependent manner in response to retinal ischemia. On the other hand, inhibiting the CaMKII activation can mitigate RGCs death both in vivo and in vitro. In the diabetic mice, CaMKII mediated RGCs death can be induced by alleviated by resveratrol, a CaMKII inhibitor [114]. Moreover, the autocamtide-2-related inhibitory peptide (AIP), a specific CaMKII inhibitor can protect the RGC-5 from cytotoxicity through inhibiting the activation of caspase-3 [27]. This finding aligns with the proposed pathogenesis of glaucoma, underscoring the crucial role of CaMKII in Epac1-induced retinal neuronal injury. In this context, further investigation is necessary to explore the downstream targets of CaMKII and their exact impacts on RGCs death. Given the intricate nature of the signaling pathways that regulate cell survival, it can be inferred that the role of CaMKII is likely to be multifaceted. Intriguingly, a recent study has shown that CaMKII activation will reduce due to RGCs axonal injury in an NMDA-induced excitotoxicity model [111], implying that CaMKII can response disparately to pathological stimuli owing to its position in different signaling pathways.

**Fig. 4:**
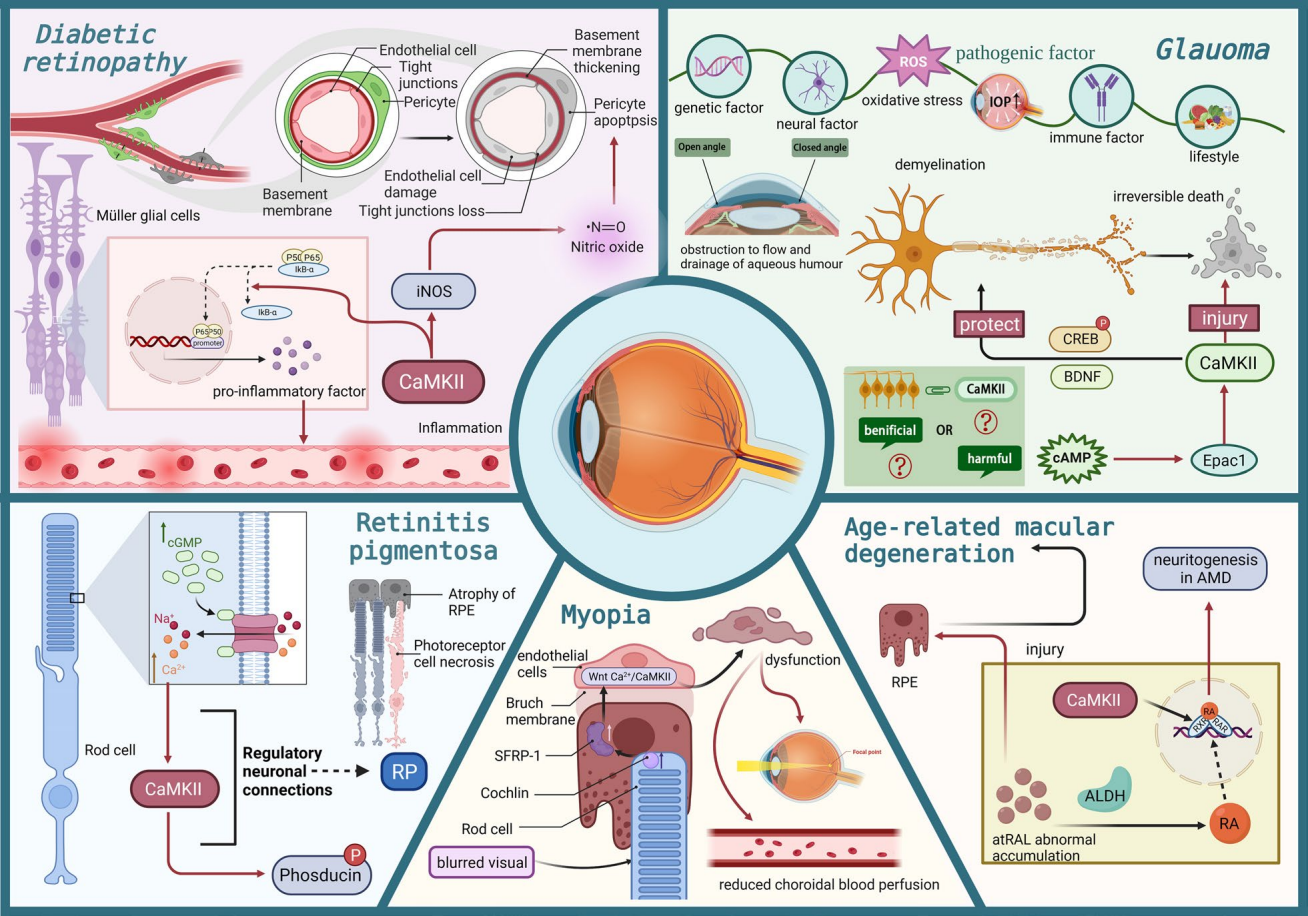
The CaMKII is involved in the pathology of eye diseases. The CaMKII activation in DR leads to pericyte apoptosis and vascular dysfunction. On the other hand, CaMKII exhibits both protective and deleterious effects on RGCs in glaucoma. CaMKII is implicated in the neurogenesis in AMD pathology. The CaMKII activation promotes the progression of myopia. CaMKII can also modulate neuronal connectivity in the RP retina

As a key downstream effector of CaMKII, the cAMP response element–binding protein (CREB) plays an important role in regulating synaptic plasticity and the survival of neurons [115]. Reactivating CaMKII and its downstream target CREB can potently protect RGCs from excitotoxicity, ameliorate the axon degeneration, and preserve the long-distance projection of RGCs axons from the retina to visual relay center in the brain. In addition, BDNF has been extensively studied and is known for its contributions to synaptic development and the formation of connections between neurons [116]. Actually, BDNF interacts with two primary receptor types: the tropomycin receptor kinase B (TrkB) and the p75 neurotrophin receptor (p75^NTR^) [117, 118]. The binding of p75^NTR^ to proBDNF can trigger apoptosis and hinder neurite growth [119]. Several recent studies have shown that some morphological changes manifest in the soma and dendrites of RGCs before axonal degeneration [120], these pathological alterations induce serious effects on synaptic efficacy, which may underlie the functional deficits prior to RGCs loss in glaucoma patients. Conversely, the BDNF-induced TrkB activation has shown promise in rescuing RGCs, in which CaMKII-related pathways are potentially involved in. In a glaucomatous mouse model, an intravitreal injection of BDNF can up-regulate the expressions of p-CaMKII and p-CREB in retina the expression of F-actin, a component of RGCs dendritic spines also increase significantly [121]. These findings suggest that CaMKII/CREB can affect the synaptic plasticity, thereby delaying the synapse degeneration of RGCs. In this context, CaMKII has similarly dual effects on RGCs as it does on RPE: under certain circumstances, CaMKII activation causes excitotoxic death of RGCs [30], whereas CaMKII also promote the survival of RGCs, in other conditions [122]. As aforementioned, CaMKII acts as the central coordinator and node signal of multiple pathways, it plays different roles in response to the upstream and downstream signals (Fig. 4). Therefore, the opposite manifestations may be ascribed to the highly context specific of CaMKII and the heterogeneous sensitivity of neurons to different pathophysiological stimuli, yet a clearer mechanism requires further research.

### Diabetic retinopathy

DR is a common microvascular complication of diabetes in the retina that acts as the leading cause of vision loss in working-age population. It is caused by the breakdown of the blood-retinal barrier (BRB) and retinal pericyte injury due to hyperglycemia associated with early diabetes [123]. Clinically, DR can be divided into two stages: non-proliferative diabetic retinopathy (NPDR) and proliferative diabetic retinopathy (PDR). NPDR stands for the early stage of DR, which involves the increased level of vascular permeability and capillary occlusion. PDR is regarded as a more advanced stage of DR that marked by neovascularization. At this stage, severe vision impairment may occur due to the vitreous hemorrhage caused by aberrant angiogenesis. In addition to abnormal neovascularization, BRB destruction can induce the fluid accumulation in the macula, ultimately leading to diabetic macular edema and even blindness [124].

Müller glia (MG) cells span the neuroretina and provide mechanical and nutritional support to the highly specialized retinal neurons. MG cells play a critical role in the pathogenesis of DR. It has been reported that MG cells induce the synthesis and secretion of a set of substances under hyperglycemia conditions, such as pro-inflammatory cytokines as well as VEGF, and the latter is a pivotal regulator of angiogenesis [125]. NF-κB is a major mediator for the transcription of these genes involved in inflammatory pathways [126]. NF-κB is composed of a p65 and p50 heterodimers that, in the classical pathway, bind to the inhibitors of kinase B-α (IkB-α), and remain inactive in the cytoplasm. IkB-α is a naturally unfolded protein with a very short half-life (a few minutes) due to the presence of a sequence called PEST at the C-terminal, which facilitates its ubiquitin-independent degradation via the proteasome [127]. When combined with NF-κB, the PEST sequence of IkB-α is shielded, thus obtaining the stability and a longer half-life. Both in vivo and in vitro studies have found the elevated NF-κB expression and reduced IkB-α expression in rat DR model. Several molecules should be responsible for the inhibitory effect on the IkB-α expression. For instance, high-mobility group box 1 protein, a well-conserved non-histone DNA-binding protein, is shown to promote the release of inflammatory cytokines [128]. The CaMKII-related pathways can induce comparable pro-inflammatory outcomes via contrasting mechanisms. Previous studies have shown that CaMKII can phosphorylate the proteasome Rpt6 subunit, thereby stimulating the turnover of IkB-α and promoting the release of p65 and p50 [129]. The released heterodimers of p65 and p50 enter the nucleus, bind to promoter sequences of pro-inflammatory genes, and induce transcription of them, such as IL-8, IL-1β, and monocyte chemoattractant protein-1. Recent evidences suggest that the CaMKII activity is essential for initiating the NF-κB mediated pro-inflammatory programs under hyperglycemia conditions [129]. On the other hand, inhibiting the activity of either CaMKII or proteasome can block the NF-κB activation and subsequent inflammatory response [129]. Inducible nitric oxide synthase (iNOS) is known to be an essential mediator in diabetic complications [130]. Several lines of evidences suggest that the presence of iNOS in the diabetic retina cause a range of detrimental consequences, including inflammation, oxidative stress, acellular capillaries, pericyte ghosts, and BRB breakdown [131, 132]. These pathological processes may be associated with the excessive production of nitric oxide (NO), a gas with diverse biological functions synthesized from arginine under the catalytic action of NOS. Under the physiological condition, constitutive isoforms of NOS, namely endothelial NOS and neuronal NOS, generate a low amount of NO, which has a positive impact on ocular hemodynamics and cell viability [133]. Conversely, iNOS is expressed exclusively in response to pathologic stimulations and then induce bursts of NO. The considerable quantity of NO has the ability to interact with various molecules, particularly superoxide, resulting in the formation of peroxynitrite. This compound can subsequently facilitate the bactericidal or cytotoxic reactions. However, excessive NO can lead to tissue injury and cell apoptosis, eventually triggering the onset of DR. Researchers propose that CaMKII serves as an upstream regulator of iNOS and plays a role in inducing pericyte death. An experiment has demonstrated that in the DR mouse model, the levels of CaMKII and iNOS increase simultaneously, leading to the apoptosis of pericytes [134]. Conversely, the intravitreal injection of AIP, a specific inhibitor of CaMKII, can alleviate the diabetic pathologies. Furthermore, CaMKII acts as an important contributor to the vascular dysfunction in DR [135]. A study has revealed that prolonged hyperglycemia induces an elevation in Ca^2+^ influx in retinal capillary endothelial cells, which subsequently triggers an upregulation of CaMKII activity [136]. Activated CaMKII evokes two downstream apoptotic pathways, namely the death-receptor pathway and the mitochondrial pathway [136]. Fas, a member of death receptor family, plays a critical role in initiating cell death. The activated CaMKII phosphorylates JNK, leading to the up-regulation of Fas [136]. The binding of Fas to its ligand can modulate the downstream caspases signaling. Additionally, CaMKII activation leads to the reduced value of mitochondrial membrane potential in a cultured macaque choroid-retinal endothelial cell line (RF/6A), which promotes the cytochrome c release from the mitochondria into the cytosol, thereby initiating the apoptosis of endothelial cells [136]. Cathepsin D (CD), an aspartic protease, has been found to disrupt the endothelial junctional barrier in the diabetic retina, leading to increased cell permeability [137]. A recent study shows that the administration of pro-CD to diabetic rats results in an elevated level of CaMKII phosphorylation, indicating that CaMKII is involved in the development of vascular dysfunction in DR [138].

### Retinitis pigmentosa

RP is an inherited retinopathy that is characterized by the gradual death of photoreceptor as well as the atrophy of RPE. This disease initially presents as nyctalopia, which is subsequently followed by the progressive decline in visual acuity until complete blindness occurs. Mutations in the gene encoding rod photoreceptor-specific cGMP phosphodiesterase 6 have been identified as the predominant etiological factor in autosomal recessive RP. The rd1 mouse equally carries a mutation in this particular gene, rendering it a frequently employed animal model for studying the human disease RP [139]. Due to the rd1 mutation, rods yield the functionally unrelated protein leading to cGMP accumulation in the outer segments. On the other hand, the regulation of cGMP-gated cation channels in rods is typically achieved through fluctuations in cGMP levels generated via the phototransduction cascade [140]. Consequently, the accumulation of cGMP in rd1 mice can elevate Ca^2+^ levels. Although abnormal levels of calcium are likely to initiate the process of RP, the downstream signaling pathways involved in the execution of photoreceptor apoptosis remain enigmatic. Recently, several transcriptomics studies have been performed to reveal the molecular and genetic mechanisms underlying retinal degeneration. For instance, the application of whole-genome and exome sequencing on a Sicilian family with a suspected form of cone-rod dystrophy led to the identification of 9 mutations that are pivotal in the clinical manifestations of the disease phenotype [141]. Furthermore, a whole exome sequencing was conducted on 17 consanguineous pedigrees of Iranian descent with inherited retinal dystrophies and researchers identified 5 novel genomic variants, thereby expanding the mutational spectrum [142]. As for other diseases, 5 patients with brain arteriovenous malformations, for instance, underwent whole exome sequencing to discover gene mutations linked to pre-existing or novel signaling pathways [143]. Transcriptomics studies can also enrich our understandings of the CaMKII in RP pathogenesis. Experimental evidences from high resolution proteomics have demonstrated that calcium-induced CaMKII overactivation of leads to a different pattern of phosducin phosphorylation in the rd1 mice [144]. Phosducin is a regulatory protein implicated in the phototransduction cascade of photoreceptors. The combination between phosducin and the β-γ subunits of transducins will promote the translocation of the complex from outer segments to inner segments. Conversely, the signal flow in the outer segment is attenuated due to the impacts of transducin, leading to a reduction in the sensitivity of light-adapted rods [145]. This procedure requires the participation of dephosphorylated phosducin. Previous research has shown that phosducin can be phosphorylated rapidly by CaMKII [146]. In this context, the phosducin in the retina of rd1 mouse remains highly phosphorylated all the time due to the CaMKII overactivation. The continuous phosphorylation of phosducin may result in dysfunctional light/dark response in the rod photoreceptors of rd1 mouse [144]. Previous studies find that the CaMKII can be influenced by the light–dark status as it is activated prominently in the dark-adapted retinas [147]. Additionally, it has been shown that the CaMKII activation is implicated in the dynamic modulation of horizontal cell dendrites in the retina of carp during dark adaptation [148]. Another experiment further illustrated the significant role of calcium modulation in the neuronal connectivity of visual system [149]. In RP models, the absence of visual inputs from photoreceptors, is known to trigger a series of retinal remodeling events. The remodeling ultimately leads to the atypical synaptic connections among retinal neurons [150]. As such, the presented evidence indicates a potential involvement of CaMKII in retinal remodeling. However, further investigations are required to validate the potential involvement of CaMKII in the pathology of RP. Thus far, several calcium channel blockers have been employed in RP models to assess their effectiveness [151]. Nevertheless, the lack of cellular steps subsequent to the administration makes it difficult to provide a comprehensive explanation for the conflicting results observed in these experiments. For instance, early studies show that diltiazem, a calcium channel blocker frequently utilized in regular therapeutic regimens, can retard the rod photoreceptor loss in rd1 mice, thus providing partial rescue of scotopic vision [152]. However, controversial evidences suggest that the diltiazem-mediated calcium blockade does not produce any protective effect on the rd1 mice or rcd1 canine [153]. In this context, it is crucial to investigate the downstream signaling following dosing and determine the potential involvement of CaMKII in this process.

### Myopia

Myopia is a prevalent disorder leading to visual impairment, and has emerged as a significant health concern among the pediatric and adolescent population [154]. If left uncontrolled, some severe cases have the potential to rapidly advance into high myopia, leading to irreversible pathological changes in the fundus. Therefore, the correction of myopia holds significant importance. Thus far, the pathogenesis of myopia remains unclear. Recently, researchers have made a pivotal discovery regarding the crucial role of blurred retinal images and diminished choroidal vascular perfusion in the occurrence of nonpathologic myopia [155]. Cochlin, an extracellular matrix protein encoded by the *coch* gene, is found in the retinal photoreceptors adjacent to the RPE. Thus far, researchers have collected a wealth of proteomics data on the myopia, thereby better clarifying the underlying pathogenesis, as well as informing subsequent preventive measures [156]. Utilizing high-throughput proteomics, a notable increase in the expression of cochlin is found in both guinea pig lens-induced myopia and form-deprived myopia models [157]. Furthermore, cochlin is responsive to the blurred visual stimuli detected by photoreceptors, leading to the upregulation of secreted frizzled-related protein 1 (SFRP-1) in RPE cells [157]. SFRP-1 can diffuse across the Bruch membrane and enter choroidal vascular endothelial cells, driven by a concentration gradient. In the choroidal vascular endothelial cells, the SFRP-1 may initiate noncanonical Wnt Ca^2+^/CaMKII signaling, thereby inducing dysfunction in these cells [157]. This dysfunction can subsequently lead to reduced choroidal blood perfusion and the occurrence of myopia. Despite the existed knowledge concerning the role of CaMKII in myopia, the specific mechanisms of downstream signaling in choroidal vascular endothelial cells remain enigmatic. Through the gene set enrichment analysis, researchers have found an active calcium signaling pathway that is involved in the development of pathological myopia. This pathway is characterized by the upregulation of *Camk2a* gene [158]. Further research on the signaling pathways associated with CaMKII can enhance our comprehension of the etiology of myopia.

### Age-related macular degeneration

AMD is a retinal degenerative disease that acts as the primary cause of irreversible blindness among the elderly population. It is characterized by aberrant functioning of RPE cells and eventual photoreceptor demise. Emerging evidences suggest that the level of levels of retinoic acid (RA) increases significantly in the light-induced AMD model [159]. As an active metabolite of vitamin, RA plays a critical role in retinal development. For instance, RA is involved in the process of neurite remodeling in carp horizontal cells under complete darkness [160]. Additionally, the elevated RA level has also been detected in the vitiligo mice with retinal degeneration [161]. In RA signaling pathway, the conversion of all-trans-retinal (atRAL) to RA is facilitated by aldehyde dehydrogenases (ALDH) [162]. It is well-established that atRAL serves as a significant intermediary in the visual cycle between RPE and photoreceptors [163]. Experimental evidence suggests that the abnormal accumulation of atRAL acts as the primary cause of RPE dysfunction, which potentially induces downstream retinal remodeling in AMD [76]. This anomalous process necessitates the binding of RA to the retinoic acid receptors (RARs) [159]. RARs frequently engage in heterodimerization with retinoid X receptors (RXRs) to establish complexes that govern the downstream gene expression. Furthermore, RXRs primarily serve as crucial auxiliary receptors that augment the signaling of numerous nuclear receptors, such as RARs [164]. Emerging evidences suggest that CaMKII is capable of modulating the RA signaling pathway. For instance, administration of KN-62, a specific CaMKII inhibitor, can promote the neuritogenesis through enhancing RXRs activity in the light induced AMD model [159]. In myeloid leukemia cells, it has been observed that KN-62 has the ability to augment the transcriptional activity of RARs [165]. These findings confirm the intricate relationship between the CaMKII and RA signaling pathways, implying that CaMKII is potentially involved in the pathologicy of AMD. Ca^2+^ signaling is also implicated in the pathology of AMD. For instance, the pre-treatment of Ca^2+^ chelator has been shown to mitigate NaIO_3_-induced epithelial–mesenchymal transition of RPE cells, a process related to pathogenesis of AMD [166]. In human RPE cells fed with bovine photoreceptor outer segments, calcium overload triggers lipofuscin formation and accumulation, a characteristic linked to the advancement of AMD [167]. Moreover, intracellular calcium signaling in ARPE-19 cells leads to the excessive production of IL-8, which is also found at elevated level in AMD patients [168]. Given the intimate connection between Ca^2+^ and CaMKII activation, further investigations are rational to elucidate the contributary role of CaMKII in the AMD.

## CaMKII antagonist with therapeutic potentials

CaMKII serves as a pivotal mediator of several cellular signaling pathways, encompassing Ca^2+^ influx, oxidative, nitrosative stress and hyperglycemia. Through the phosphorylation of a wide range of downstream targets, CaMKII effectively transduces these signals into both physiological and pathological cellular responses, thereby exerting regulatory effects on the intracellular Ca^2+^ dynamics, contractility, and metabolism [169]. In this context, CaMKII may acts as an attractive therapeutic target for ocular diseases. At present, many therapeutic strategies have been developed, including small molecules, peptides, antisense oligodeoxynucleotides (ASO), and targeting the CaMKII signal transduction (Table 2). Its application in organs such as heart and brain are relatively mature. For instance, in a rat model of middle cerebral artery occlusion, CaMKII activation can be intervened by small interfering RNA [170]. Moreover, during the ischemic stroke caused by excessive glutamate, CaM-KIIN, a natural CaMKII inhibitor, can curb the CaMKII over-activation, and provide therapeutically neuroprotection after excitotoxicity [171].

**Table 2:** Principles, advantages and disadvantages of various CaMKII targeting strategies

Types	Examples	Principle	Advantages	Disadvantages
Small molecules	KN-93	CaM antagonist	• High bioavailability • Rapid induction • Cell permeant • Have reasonable half-lives	• Inhibition of extracardiac CaMKII • Reduced kinase and isoform specificity • Low potency • Off-target effects
	AS105	Inhibit CaMKIIδ		
	RA306	Inhibit CaMKIIδ and CaMKIIγ		
	Rimacalib	/		
	NP202	Inhibition of CaMKII in vitro		
Peptides	CaM-KIIN derivatives	Derived from two endogenous genes encoding CaMKII inhibitory peptides	• Genetically encoded • Potent and selective • Specificity for CaMKII	• Intractable delivery • Poor bioavailability
	Pseudosubstrate mimetics	Derived from CaMKII’s self-regulatory domain		
ASOs	/	Pairing with the base of messenger ribonucleic acid	• Long plasma half-life • Isoform and splice specificity	• Slow induction
Indirect targeting	Phosphatases	Inhibit PTMs	• Avoids CaMKII-related • Off-target effects	• Likely incomplete blockade • Lack of selectivity
	CaM-KAP	recruits CaMKII to the SR		

### KN-93

**Fig. 5:**
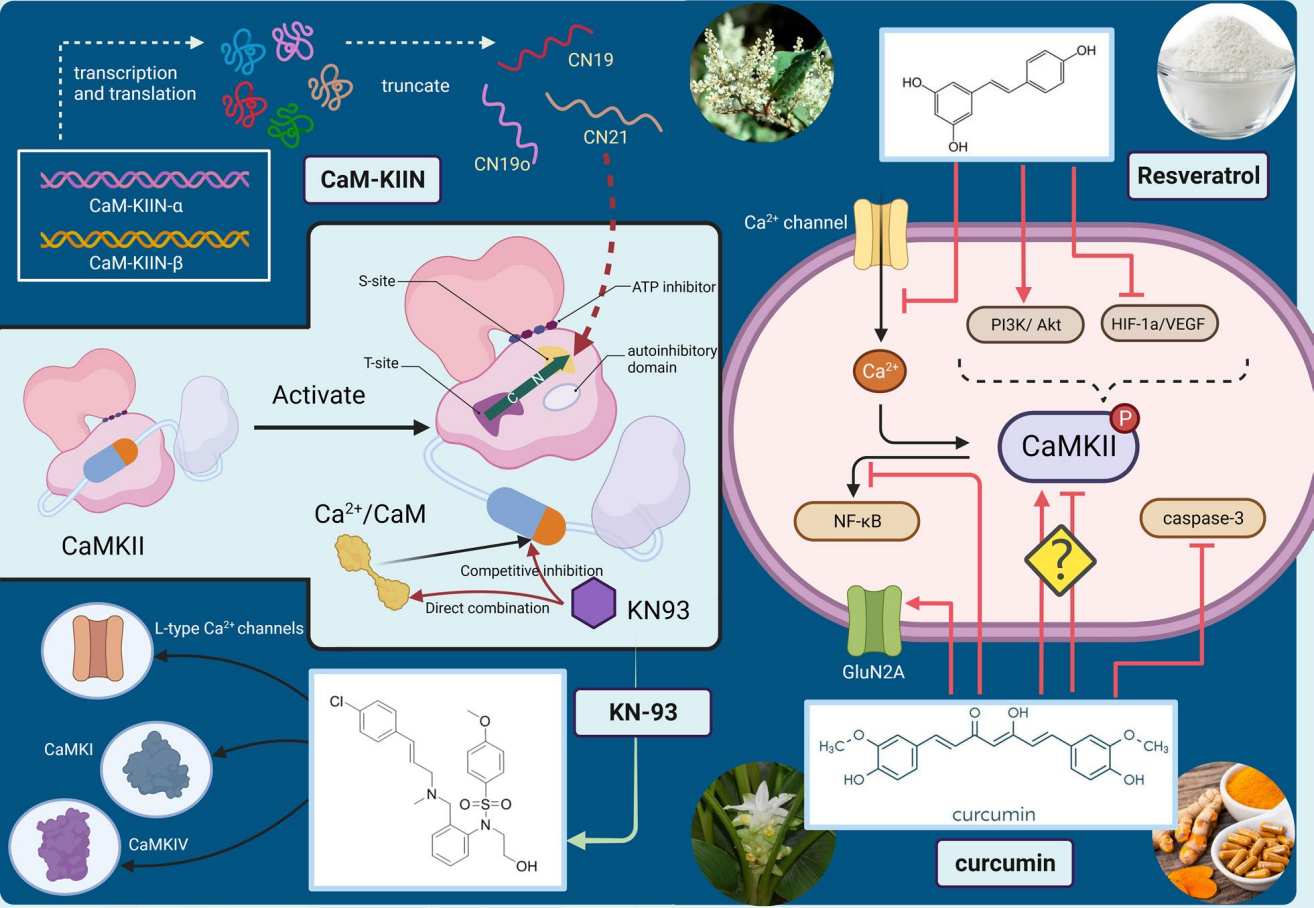
The mechanism of CaMKII inhibitors. KN93 inhibits CaMKII through competing with Ca^2+^/CaM for the kinase or directly binding to Ca^2+^/CaM. CaM-KIIN interacts with the T-site of CaMKII to achieve inhibitory effects. The mechanism of plant-derived inhibitors has not been elucidated clearly

KN93, a specific CaMKII inhibitor, can reversibly inhibit CaMKII function through competing with Ca^2+^/CaM for the kinase. KN93 contains three hydrophobic rings, which are structurally resemble hydrophobic side chains of amino acids that CaM often interacts with Ca^2+^. Some research also shows that KN93 inhibits CaMKII activation through directly binding to Ca^2+^/CaM (Fig. 5) [172]. Due to its membrane-penetrating properties, it has been used as a potent inhibitor of CaMKII activation to promote cell survival. For instance, KN93 alleviates the blue light-induced mitochondrial rupture and AIF-mediated necroptosis in retinal R28 cells through blocking the CaMKII-Drp1 pathway [82]. Moreover, KN93 can block the activation of CaMKII/NF-κB signaling induced by hyperglycemia [135]. In a glutamate-induced retinal neuronal RN, the CaMKII inactivation induced by KN-93 can alleviate the glutamate-induced retinal damages [87]. It is demonstrated that VEGF-induced phosphorylation of Akt is dependent on the activation of CaMKII. and KN93 is effective to inhibit this proangiogenic process in a vitro model [20]. Additionally, researchers find that KN93 can inhibit the uptake of [^3^H] adenosine and the transporter-mediated release of purines in cultured avian retinal cells [173]. KN93 has been shown to inhibit various subtypes of CaMKII: it can lead to a reduced hyperalgesia level in rat models with diabetic neuropathic pain through downregulating the expression of p-CaMKIIα in the surface layer of the spinal dorsal horn [174]. Additionally, KN93 has been found to inhibit CaMKIIδ in malignant mesothelioma and acral melanoma, suggesting its potentials as a candidate for molecular targeted anticancer drugs [175]. However, once the CaMKII is activated and undergoes automatic phosphorylation, the KN-93 mediated inhibitory effect on CaMKII is lost. This is also observed when CaMKII is autonomously activated through oxidation or GlcN acylation [176]. KN-93 has the disadvantages such as the low efficiency and poor specificity. L-type Ca^2+^ channels, the rapid component of delayed rectifier potassium current, and other kinases, such as CaMKI and CaMKIV, have also been shown to be targets for KN-93 [177, 178]. This indiscriminate inhibition makes this type of CaMKII inhibitors difficult to be used on humans. KN93 is commonly utilized as an experimental reagent to assess the impact of various drugs or genes on CaMKII. In a recent study, KN93 was found to counteract the up-regulating effect of Jiawei-Xiaoyao pill (JWX), a traditional Chinese medicine, laying the groundwork for JWX to induce rapid antidepressant effects through activating the CaMKII signaling pathway [179].

### CaM-KIIN

Polypeptide inhibitors can be divided into the CaM-KIIN derivatives and the pseudosubstrate mimics according to their source. CaM-KIIN is a natural, specific CaMKII inhibitor that encoded by two endogenous genes called CaM-KIIN-α and CaM-KIIN-β (CAMK2N2/CAMK2N1) [51, 180]. A recent study shows that curcumin is the first human-derived, endogenous, nonchemical inhibitor of CaMKII. CaM-KIIN can inhibit all CaMKII subtypes with an IC_50_ value as low as 50 nm [181]. The peptides have been optimized and truncated into the smallest functional units to develop CN21, CN19 and CN19o, which are more potent and selective [169]. CN21 is the minimal region of CaM-KIIN that retains full potency and specificity of CaMKII inhibition [182]. On the other hand, CN19, produced through the truncation of the end of CN21 by 2 amino acids, contains the minimal inhibitory region with full inhibitory potency [171]. Compared to CN19, CN19o inhibited CaMKII with 250 folds enhanced potency. CN21 and CN19o have been leveraged into cell-permeable variants by fusion with the Tat peptide. TatCN21 can inhibit filopodia motility and insulin secretion [182]. And TatCN19o has been shown to alleviate ischemic injury in the brain [183]. Previous researchers have found that CaM-KIIN interacts with the T-site of CaMKII to achieve inhibitory effects [182]. Through connecting with the noncatalytic T-site of CaMKII, CaM-KIIN prevents the substrate access to the immediately adjacent catalytic S-site (Fig. 5). CN21 blocks efficiently the substrate- and T305 autophosphorylation of CaMKII, but surprisingly it only mildly affects the T286 autophosphorylation [182]. CaM-KIIN is competitive with the region around T286, and strengthens the CaM binding required for presentation of T286 as a substrate [182]. GluN2B is another CaMKII substrate known to bind to the T-site. CN21 can block GluN2B by competitive inhibition [184]. CaM-KIIN expression is upregulated during the consolidation of fear memory, suggesting that it may be involved in the higher brain function. Additionally, a transfection experiment using LoVo cells reveals that CaM-KIIN induces inhibitory effects on cell proliferation [185].

Both KN inhibitors and CaM-KIIN offer protection against the glutamate induced excitotoxicity. However, only the CaM-KIIN applied post-injury could be therapeutically effective. Compared with KN inhibitors, CaM-KIIN provides a promising alternative because it potently inhibits CaMKII but not CaMKI, CaMKIV, PKA, or PKC [182]. The Tat fusion peptides, particularly noted for their CaMKII-selective nature and enhanced efficacy, have demonstrated utility in both in vitro and in vivo settings, including the ability to traverse the blood–brain barrier [185]. Nevertheless, the limited bioavailability and intricate nature of CaM-KIIN have hindered its widespread application compared with KN inhibitors, particularly within the realm of ophthalmology. Currently, the CaM-KIIN are predominantly used to treat neurological disorders. Anyway, CaM-KIIN still provides a powerful and potential tool for studying cellular CaMKII function.

### Plant-derived inhibitors

Natural products derived from plants have the advantages of pleiotropic activity, safety, and ease of accessibility [186]. For instance, resveratrol is a natural polyphenolic phytoalexin with a range of beneficial effects. Resveratrol has been shown to exert neuroprotective effects on the optic neuritis and multiple sclerosis mouse model [187]. CaMKII activation is involved in the demise of retinal neurons in a self-phosphorylation-dependent manner [188]. It has demonstrated that resveratrol can inhibit CaMKII activation, thus reducing the RGCs death in diabetes mouse model. In greater detail, resveratrol might inhibit CaMKII activity through blocking a calcium- and voltage-gated calcium channel–dependent process [189]. In a model of retinal I/R injury, resveratrol has been shown to mitigate the RGCs loss and visual impairments through inhibiting the HIF-1a/VEGF pathway which is closely correlated with CaMKII [114]. The researchers speculate that the resveratrol mediated protection may achieved through inhibiting CaMKII activation. However, it is important to note that CaMKII is not the sole target of resveratrol in the retina. For instance, low-dose trans-resveratrol has been shown to mitigate diabetes-induced RGCs degeneration through the TyrRS/c-Jun pathway [190]. Additionally, resveratrol can activate sirtuin 1, a deacetylase known to protect against various ocular diseases [191]. A recent study has combined the resveratrol with nanotherapeutics to improve the therapeutic effects on macular degeneration, implying promising prospects for future development [192].

Another widely studied plant-derived inhibitor is curcumin, a natural flavonoid component found in the rhizome of curcuma longa. Many studies have reported that curcumin protects retinal neuron cultures from glutamate toxicity [193]. It has been shown that GluN2B plays a critical role in the excitotoxic cell death, while GluN2A is implicated in the curcumin mediated neuroprotection [193]. Curcumin is able to protect rat retinal neurons against NMDA-induced excitotoxicity through increasing the expression of GluN2A-containing NMDA receptor. Moreover, the curcumin induced GluN2A expression largely depends on CaMKII phosphorylation [194]. However, some researchers also propose that the neuroprotective effect of curcumin is through inhibiting rather than promoting of CaMKII activity. For example, in a streptozotocin (STZ)-induced diabetic rat model, curcumin attenuates diabetes-induced photoreceptor apoptosis through reducing the cleaved caspase-3 expression and inhibiting CaMKII activity [135]. Another study also shows that curcumin inhibits the CaMKII/NF-κB signaling pathway, thereby reducing the release of inflammatory mediators such as VEGF, iNOS and ICAM-1 in the STZ induced diabetic rats [135]. Additionally, curcumin has been found to mitigate hydroxychloroquine-induced apoptosis and oxidative stress through inhibiting transient receptor potential melastatin 2 channel signaling pathways in RPE [195]. Recent study has incorporated the curcumin-loaded polydopamine nanoparticles into the GelCA hydrogel matrix to create a multifunctional nanocomposite hydrogel. This nanocomposite hydrogel demonstrated satisfactory compatibility both in vitro and in vivo [196].

## Conclusions

Long-term investigations have consistently demonstrated the involvement of CaMKII in multiple signal transduction pathways. Extensive knowledge regarding its molecular composition and subtypes has been acquired by researchers. The CaMKII exhibit a broad spectrum of functions within the cardiac, retinal, and cerebral domains. Present investigative endeavors have predominantly concentrated on elucidating the impact of CaMKII in cardiovascular ailments, such as heart failure, arrhythmia, and cardiac ischemia and perfusion. However, researches pertaining to CaMKII in other bodily organs remain relatively scarce. This review firstly provides a thorough exposition of the role of CaMKII in ocular physiology, encompassing its molecular architecture, functional attributes and metabolism. Moreover, we elucidate the contributory role of CaMKII in the pathological mechanisms of ophthalmic diseases, such as glaucoma, DR, and RP. The exploration of CaMKII and its downstream target signals has propelled the advancement of CaMKII inhibitors. This article presents various types of CaMKII inhibitors along with their respective merits and drawbacks. Although several CaMKII inhibitors have shown their safety and effectiveness in animal experiments, their clinical profile is scarce. Thus, the preclinical proof-of-concept testing and subsequent clinical trials are necessary. Currently, several pathologic and pharmacologic questions remain to be resolved. The exact mechanism underlying the CaMKII related retinal degeneration is not clear enough. Moreover, more endeavors should be made to develop efficacious treatment approaches through experimental investigations. In view of these critical gaps, we propose specific avenues for future study. Advanced clinical trials of CaMKII should be performed to elucidate the dual roles of CaMKII. Under which conditions CaMKII may cause damage to retinal neurons, and under which circumstances it can provide protection. Since CaMKII is extensively expressed in multiple cell types, inhibition of CaMKII or its downstream reactions may produce some unnecessary side effects. Future endeavors are required to identify some effective and specific inhibitors or inhibition techniques to mitigating the adverse effects. After continuous research and exploration, enhanced comprehension of CaMKII may ultimately render it a promising therapeutic target in forthcoming times.

## Data Availability

Not applicable.

## Abbreviations

CaMKII: Ca^2+^/calmodulin-dependent protein kinase II
ox-CaMKII: Oxidation CaMKII
DR: Diabetic retinopathy
AMD: Age-related macular degeneration
RP: Retinitis pigmentosa
Ser: Serine
Thr: Threonine
T286: Threonine 286
PT286: The autophosphorylation at T286
VEGF: Vascular endothelial growth factor
OIR: Oxygen-induced retinopathy
RGCs: Retinal ganglion cells
NMDA: N-methyl-D-aspartate
BDNF: Brain-derived neurotrophic factor
Bcl-2: B cell lymphoma-2
I/R: Ischemia–reperfusion
PTMs: Posttranslational modifications
PP: Protein phosphatases
PP2A: Protein phosphatase 2A
ROS: Reactive oxygen species
ncRNA: Non-coding RNA
lncRNA: Long ncRNA
IP_3_: Inositol 1,4,5-trisphosphate
PLC: Phospholipase C
STAT3: Transcription factor 3
IL-6: Interleukin 6
SOCS3: Suppressor of cytokine signaling 3
VM: Vasculogenic mimicry
VE: Vascular endothelial
EphA2: Ephrin type-A receptor 2
RPE: Retinal pigment epithelium
AMPK: Adenosine 5′-monophosphate-activated protein kinase
Drp1: Dynamin-related protein 1
AIF: Apoptosis-inducing factor
GluRs: Glutamate receptors
Pin1: Peptidyl-prolyl isomerase 1
CAST: Calpastatin
RN: Regulated necrosis
CCBs: Calcium channel blockers
SNAP-25: Synaptosomal-associated protein 25 kDa
PKA: Protein kinase A
Epac: Exchange proteins activated by cAMP
AIP: Autocamtide-2-related inhibitory peptide
CREB: CAMP response element–binding protein
TrkB: Tropomycin receptor kinase B
p75^NTR^: P75 neurotrophin receptor
BRB: Blood-retinal barrier
NPDR: Non-proliferative diabetic retinopathy
PDR: Proliferative diabetic retinopathy
MG: Müller glia
IkB-α: Kinase B-α
iNOS: Inducible nitric oxide synthase
NO: Nitric oxide
JNK: C-Jun N-terminal kinase
CD: Cathepsin D
SFRP-1: Secreted frizzled-related protein 1
RA: Retinoic acid
atRAL: All-trans-retinal
RARs: Retinoic acid receptors
RXRs: Retinoid X receptors
ASO: Antisense oligodeoxynucleotides
STZ: Streptozotocin
JWX: Jiawei-Xiaoyao pill
